# Antioxidant and Functional Properties of Collagen Hydrolysates from Spanish Mackerel Skin as Influenced by Average Molecular Weight

**DOI:** 10.3390/molecules190811211

**Published:** 2014-07-31

**Authors:** Chang-Feng Chi, Zi-Hao Cao, Bin Wang, Fa-Yuan Hu, Zhong-Rui Li, Bin Zhang

**Affiliations:** 1 National Engineering Research Center of Marine Facilities Aquaculture, School of Marine Science and Technology, Zhejiang Ocean University, 1st Haidanan Road, Changzhi Island, Lincheng, Zhoushan 316000, China; E-Mails: expandable1@126.com (Z.-H.C.); moonriveryue@163.com (F.-Y.H.); 2 Zhejiang Provincial Engineering Technology Research Center of Marine Biomedical Products, School of Food and Pharmacy, Zhejiang Ocean University, 1st Haidanan Road, Changzhi Island, Lincheng, Zhoushan 316000, China; E-Mail: zhangbin_ouc@163.com; 3 Division of Life Science, Hong Kong University of Science and Technology, Clear Water Bay, Kowloon, Hong Kong, China; E-Mail: zlibb@ust.hk

**Keywords:** Spanish mackerel (*Scomberomorous niphonius*), collagen hydrolysate, average molecular weight (AMW), functional property

## Abstract

In the current study, the relationships between functional properties and average molecular weight (AMW) of collagen hydrolysates from Spanish mackerel (*Scomberomorous niphonius*) skin were researched. Seven hydrolysate fractions (5.04 ≤ AMW ≤ 47.82 kDa) from collagen of Spanish mackerel skin were obtained through the processes of acid extraction, proteolysis, and fractionation using gel filtration chromatography. The physicochemical properties of the collagen hydrolysate fractions were studied by sodium dodecyl sulfate polyacrylamide gel electrophoresis (SDS-PAGE), gel filtration chromatography, scanning electron microscope (SEM) and Fourier transform infrared spectroscopy (FTIR). The results indicated that there was an inverse relationship between the antioxidant activities and the logarithm of the AMW of the hydrolysate fractions in the tested AMW range. However, the reduction of AMW significantly enhanced the solubility of the hydrolysate fractions, and a similar AMW decrease of the hydrolysate fractions negatively affected the emulsifying and foaming capacities. This presented as a positive correlation between the logarithm of AMW and emulsion stability index, emulsifying activity index, foam stability, and foam capacity. Therefore, these collagen hydrolysates with excellent antioxidant activities or good functionalities as emulsifiers could be obtained by controlling the effect of the digestion process on the AMW of the resultant hydrolysates.

## 1. Introduction

At present, enormous amounts of protein-rich by-products, accounting for 50%–70% of the raw material, are produced in the process of aquatic product processing in China [1,2,3], and effective use of these by-products is an optimal method for protecting the ecological environment and manufacturing high added-value products to augment the income of seafood processors. Hence, many studies have been conducted to extract and screen the potential industrial applications of collagens from fish by-products, such as skins of unicorn leatherjacket (*Aluterus monocerous*) [4], Amur sturgeon (*Acipenser schrenckii*) [5,6], hammerhead shark (*Sphyrna lewini*) [7], sailfish (*Istiophorus platypterus*) [8], balloon fish (*Diodon holocanthus*) [9], and bighead carp (*Hypophthalmichthys nobilis*) [10]. By developing enzyme technologies for protein modification, protein functional properties could be modified and improved to be suited to serve as valuable food ingredients and industrial products [11,12,13]. It has been reported that protein hydrolysates of seafoods and their by-products, such as tilapia (*Oreochromis niloticus*) [14], croceine croaker (*Pseudosciaena crocea*) [15], salmon [16], yellow stripe trevally (*Selaroides leptolepis*) [17], monkfish (*Lophius litulon*) [18], and Tilapia (*Oreochromis niloticus*) [19], have different functional properties and great potential for nutritional and pharmaceutical applications [20]. However, these hydrolysates differed significantly from their parent proteins in structure, molecular weight (MW), nutritive values, bioactivities, and functional properties, including solubility, emulsification, and foaming ability [21].

It is well known that types of enzymes and enzymolysis conditions, including enzyme/substrate ratio, material/solvent ratio, temperature, time, and pH, could influence polypeptide chain lengths and functional properties of fish protein hydrolysates [22]. Hence, a proteolysis process under controllable conditions has been applied to improve and enhance the bioactivities and functional properties of protein hydrolysates [23]. However, an inappropriate proteolysis process could generate adverse effects on the functional properties. Therefore, an appropriate parameter is imperative to control the enzymatic hydrolysis reaction for generating the desired hydrolysates with excellent functionalities [24]. Based on previous reports [23,24,25], the average molecular weight (AMW) is one of the key factors affecting the biological and functional properties of hydrolysates and revealing the correlations between AMW and functional properties, which is crucial for the utilization of hydrolysates.

Hydrolysates of acid soluble collagen (ASC) were prepared from Spanish mackerel (*Scomberomorous niphonius*) skin in our preliminary experiment [26]. In the present text, seven hydrolysate fractions with a wide-range AMW were prepared from collagen hydrolysate of Spanish mackerel skin by using gel filtration column, and the influence of AMW on their physicochemical and functional properties was investigated.

## 2. Results and Discussion

### 2.1. SDS-PAGE Pattern of ASC from Spanish Mackerel Skin (ASC-SM)

**Figure 1:**
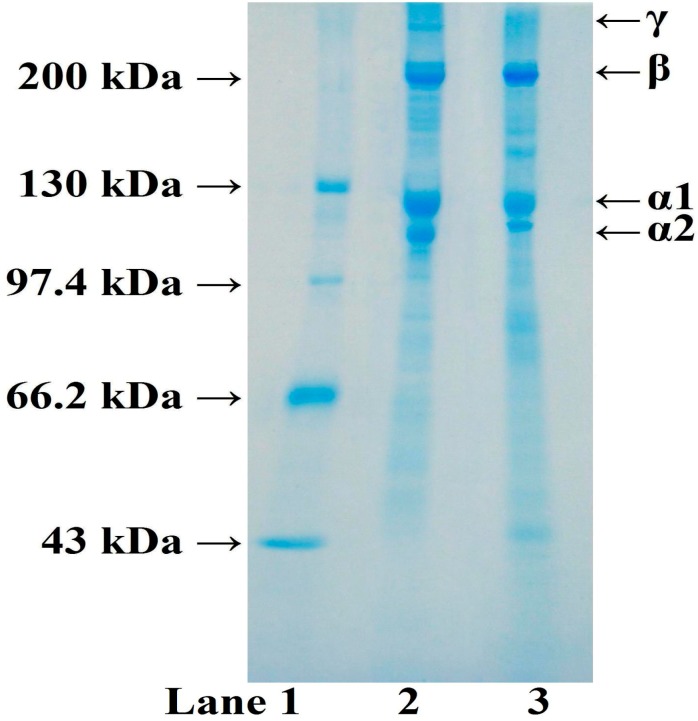
Sodium dodecyl sulfate polyacrylamide gel electrophoresis (SDS-PAGE) pattern of acid soluble collagen-Spanish mackarel (ASC-SM) on 7.5% separating gel. Lane 1: High molecular weight (MW)protein markers; lane 2: ASC-SM; lane 3: Type I collagen from calf skin.

The yield of ASC-Spanish mackarel (SM) was 13.68% ± 0.35% on the wet skin weight. Sodium dodecyl sulfate polyacrylamide gel electrophoresis (SDS-PAGE) (Figure 1) showed that ASC-SM consisted of both α1 and α2 chains, and the band of α1-chain was about twice as intense as that of α2-chain. Other cross-linked subunits with high MW, such as β-chain (dimers of α1-chains) and γ-chain (trimers of α1-chains), were also observed in ASC-SM. On the basis of subunit composition and SDS-PAGE pattern, it could be speculated that ASC-SM was mainly composed of type I collagen, which was same as the type I collagen derived from calf skin, and skins and bones of most fish [1,27,28]. It has been proven that the major component of type I collagen is composed of two α1-chains and one α2-chain ([α1]_2_α2). However, another heterotrimer (α1α2α3) was found to be a component of type I collagen of bony fish [29]. Alpha3-chain with a similar MW to α1-chain could not be effectively separated from α1-chain under the current experimental conditions, and it might be present in ASC-SM.

### 2.2. Preparation and Proximate Analysis of Fractions from ASC-SM Hydrolysate

**Figure 2:**
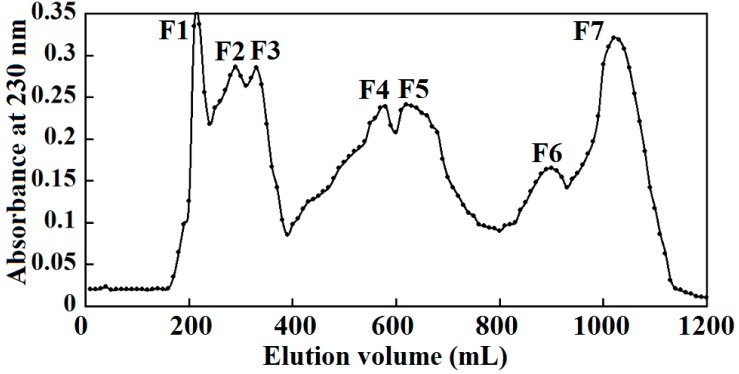
Elution profile of ASC-SM hydrolysate prepared with gel filtration on Sephadex G-100 column.

Many researches have demonstrated that hydrolysates from collagen and gelatin exhibited favorable functional properties and biological activities, which had become a topic of great interest for health food and processing/preservation industries [15,21]. In the experiment, ASC-SM hydrolysate was prepared using pepsin (2500–3500 U/mg protein, Sigma-Aldrich (Shanghai) Trading Co., Ltd.) for 5 h at 37 °C, pH 2.5, and the resulting hydrolysate (5 mL, 10 mg/mL) was fractionated through gel filtration on Sephadex G-100 column each time (Figure 2). Seven fractions (designated as F1 to F7) with different MW eluting from a gel filtration column were collected and measured at 230 nm, and the same fractions were pooled, concentrated and lyophilized.

Table 1 shows the chemical compositions of ASC-SM, ASC-SM hydrolysate, and its fractions. The ash, fat, and carbohydrate contents of ASC-SM and its hydrolysate were significantly higher than those of the seven fractions. Conversely, the protein contents of ASC-SM and its hydrolysate were significantly lower than those of the seven fractions, and the high protein content of the seven fractions was a result of the removal of most lipid and other impurities after hydrolysis and gel filtration chromatography. High carbohydrate ratios might positively impact the antioxidant activities and foam characteristics of hydrolysates [24], but the carbohydrate contents of ASC-SM, ASC-SM hydrolysate, and fractions were lower than 0.84%, so the influence could be negligible in the test. Therefore, the protein could be deemed to the major contributor to the functional properties for all the samples.

**Table 1:** The proximate compositions (%) and imino acid contents (expressed as residues/1000 residues) of ASC-SM, its hydrolysate, and seven fractions (F1 to F7).

	Moisture	Ash	Fat	Protein	Carbohydrate
ASC-SM	0.72 ± 0.02	10.58 ± 0.54	10.33 ± 0.46	78.37 ± 1.73	0.84 ± 0.04
ASC-SM hydrolysate	0.75 ± 0.05	8.48 ± 0.38	8.57 ± 0.37	81.05 ± 2.24	0.75 ± 0.06
F1	0.85 ± 0.03	4.75 ± 0.32	2.88 ± 0.15	92.74 ± 0.84	0.54 ± 0.04
F2	0.79 ± 0.03	3.65 ± 0.36	1.65 ± 0.31	94.18 ± 0.76	0.41 ± 0.06
F3	0.95 ± 0.04	3.51 ± 0.35	1.79 ± 0.23	94.51 ± 1.25	0.45 ± 0.02
F4	1.21 ± 0.02	4.02 ± 0.28	1.68 ± 0.28	94.80 ± 0.27	0.37 ± 0.04
F5	1.56 ± 0.04	4.41 ± 0.31	1.93 ± 0.18	95.14 ± 0.87	0.31 ± 0.05
F6	1.35 ± 0.02	3.81 ± 0.20	1.42 ± 0.16	95.37 ± 1.01	0.34 ± 0.05
F7	1.08 ± 0.04	4.06 ± 0.27	1.36 ± 0.25	95.39 ± 0.62	0.36 ± 0.03

In addition, the imino acid (Pro and Hyp) contents of ASC-SM, its hydrolysate, and seven fractions (F1 to F7) was detected and is shown in Table 2. Compared with the imino acid contents of ASC-SM, there were little changes in the amino acid composition. The result was consistent with the previous report that hydrolysis did not change the amino acid composition of collagen/gelatin [21]. Moreover, there were some differences in the imino acid contents of the seven fractions, but they still contributed to a high proportion of the contents, and this finding should be associated with the structure of collagen. It has been reported that all members of the collagen family are characterized by domains with repetitions of the proline-rich tripeptides, Gly-X-Y, involved in the formation of the triple helix, except for the first 14 amino acid residues from the N-terminus and the first 10 amino acid residues from the *C*-terminus of the collagen molecules, where X is generally proline and Y is mainly hydroxyproline [2,7,26]. The present results indicate that the enzyme might undermine part of the structure of collagen and change the length of the polypeptide chain, but the proline-rich tripeptides (Gly-X-Y) could still be the main components of peptides in the seven hydrolysate fractions.

**Table 2:** Imino acid contents (expressed as residues/1000 residues) and average molecular weights (AMW) of ASC-SM, its hydrolysate, and seven fractions (F1 to F7).

	Imino Acid Contents	AMW
ASC-SM	177.1 ± 1.05	----
ASC-SM hydrolysate	176.8 ± 1.37	23.75 kDa
F1	173.5 ± 1.44	47.82 kDa
F2	167.2 ± 0.91	28.77 kDa
F3	177.4 ± 1.26	26.70 kD
F4	163.3 ± 1.15	21.03 kDa
F5	168.4 ± 0.73	19.82 kDa
F6	171.1 ± 1.24	14.39 kDa
F7	165.1 ± 1.17	5.04 kDa

----:No detected.

### 2.3. Distribution of MW and AMW of ASC-SM Hydrolysate Fractions

**Figure 3:**
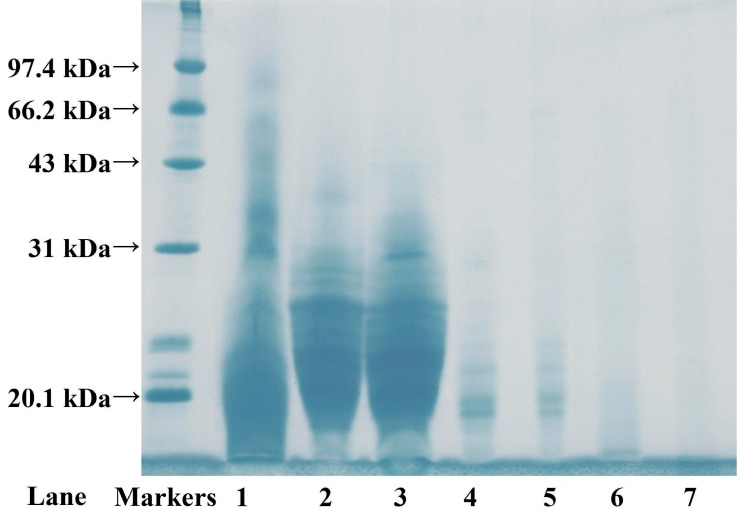
SDS-PAGE pattern of seven hydrolysate fractions from ASC-SM on 7.5% separation gel. Lane 1: F1; lane 2: F2; lane 3: F3; lane 4:F4; lane 5:F5; lane 6:F6; lane 7:F7.

Figure 3 shows the composition distribution of the ASC-SM hydrolysate fractions (F1 to F7). The AMWs of seven fractions were determined by high pressure size exclusion chromatography (HPSEC) on a TSK-G3000SW_XL_ in order to illuminate the interrelations between AMWs and their functional properties. According to the AMW values, the seven hydrolysate fractions of ASC-SM are listed in a descending order as follows: F1 (47.82 kDa) > F2 (28.77 kDa) > F3 (26.70 kDa) > F4 (21.03 kDa) > F5 (19.82 kDa) > F6 (14.39 kDa) > F7 (5.04 kDa) (Table 2), and the result indicate that the fractions are eluted out with theoretical AMWs according to the theory of a gel filtration chromatogram. However, the result was slightly different from that of the SDS-PAGE patterns of the hydrolysate fractions of ASC-SM. Rath *et al.* reported that some proteins with greater hydrophobic contents, for instance membrane proteins, were intrinsically harder to treat accurately using a chemical denaturant (SDS) to remove their structure and turn the molecule into an unstructured linear chain [30]. Hence, the MWs of those proteins determined using the SDS-PAGE method were different with their actual MWs. In the test, the result of SDS-PAGE analysis of the MWs of F1, F2, and F3 might have been in disagreement with the result of gel filtration chromatography analysis for the same reasons.

### 2.4. Ultrastructure

**Figure 4:**
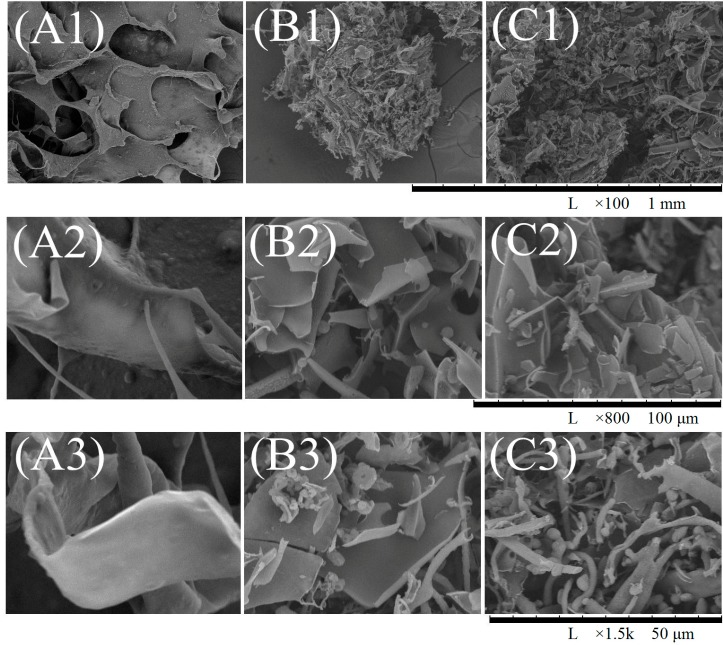
Scanning electron microscopic (SEM) images of ASC-SM (**A**); F1 (**B**) and F7 (**C**) with magnifications of ×100 (1), ×800 (2), and ×1.5 K (3).

Zhang *et al.* reported that freeze-drying was the optimal method to prepare homogeneous multihole collagen and the best way to maintain the original state of the sample [31]. Figure 4 indicates that scanning electron microscopic (SEM) pictures of lyophilized ASC-SM, F1, and F7 show obvious differences at the same lyophilization conditions and concentrations.

As shown in Figure 4A, the lyophilized ASC-SM has a schistose structure and remains as an integral whole, indicating that ASC-SM maintain a structural integrity. F1 has the highest AMW (47.82 kDa) among the seven hydrolysate fractions, and Figure 4B shows the SEM pictures of F1 with different magnifications, which illustrates that schistose collagen has partially been broken down into small particles and slices after pepsin hydrolysis. Based on the size of particles and slices, the components in F1 can roughly be classified into two types: a bigger type, which is analogous to the sheet structure with a mean length larger than 50 μm and a smaller type, which is similar to a particle and has a rod-like shape with a mean length of less than 25 μm. Among these seven hydrolysate fractions, F7 has the lowest AMW (5.04 kDa). As shown in Figure 4C, F7 presents particles with an even surface and some small slices, and all of them have a mean particle length ranging from 5 to 10 μm, which might be due to their high hydrolysis degree.

### 2.5. Fourier Transform Infrared Spectroscopy (FTIR) Analysis

**Figure 5:**
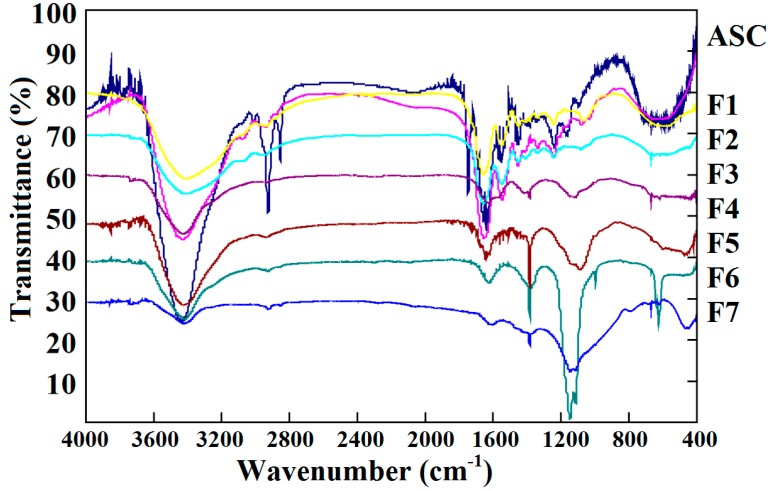
Fourier transform infrared spectroscopy (FTIR) spectra of ASC-SM and seven hydrolysate fractions.

Figure 5 shows the infrared (IR) spectra of ASC-SM and its hydrolysate fractions. The typical characters for type I collagen, including five peaks (amide A (3433 cm^−1^), amide B (2926 cm^−1^), amide I (1641 cm^−1^), amide II (1549 cm^−1^) and amide III 1240 (cm^−1^)) are observed in the spectrogram of ASC-SM. Amide III represents the combination peaks between C-N stretching vibrations and N-H deformation from amide linkages as well as absorptions arising from wagging vibrations from CH_2_ groups of the glycine backbone and proline side-chains, and the wave number of 1454 cm^−1^ represents the existence of a C-H bending vibration, and the absorption ratios between amide III and the peak of C-H bending vibration demonstrate the presence of a helical structure [32,33]. The absorption ratio between amide III and the 1456 cm^−1^ band is close to 1.0 in the spectrogram of ASC-SM (Figure 5), and the result indicates the presence of triple-helical structure.

Compared with ASC-SM, a difference in the spectra of the seven hydrolysate fractions can be observed. For instance, the IR spectrum of F7 (AMW 5.04 kDa) totally differs from that of ASC-SM. In addition, the bands of 1456 cm^−1^ and amide III fade away, which confirms that the triple-helical structure of F7 disappears after pepsin hydrolysis.

### 2.6. Antioxidant Activity

In the present research, 2,2-diphenylpicrylhydrazyl (DPPH)^•^ and hydroxyl (HO)^•^ scavenging activities and the reducing power of seven fractions of ASC-SM hydrolysate were determined and used to evaluate their abilities to protect against oxidation of the samples.

**Figure 6:**
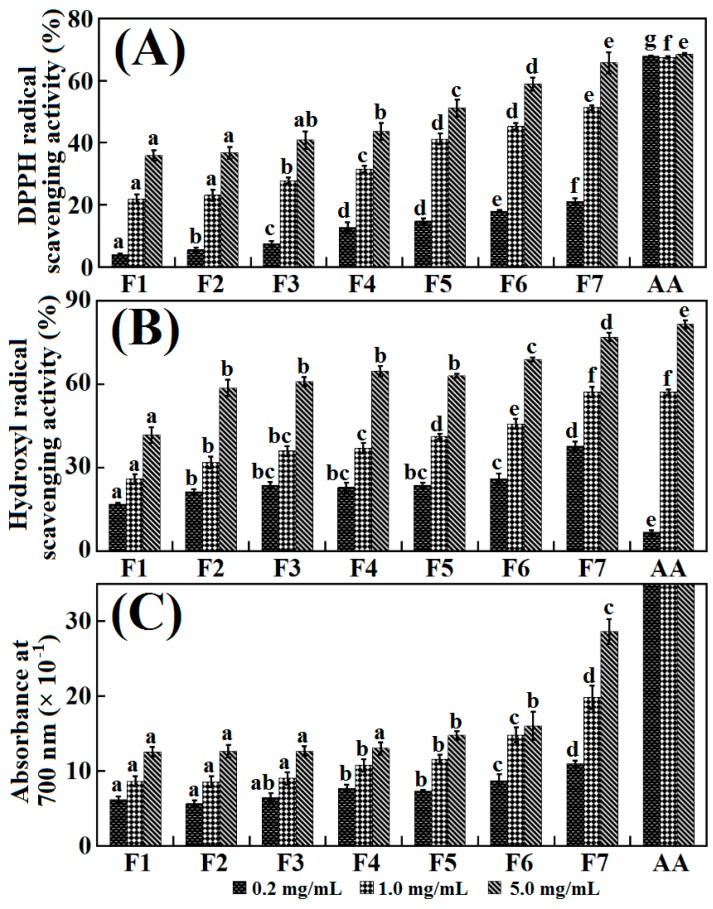
2,2-Diphenylpicrylhydrazyl (DPPH)^•^ (**A**) and hydroxyl (HO)^•^ (**B**) scavenging activities, and reducing power (**C**) of seven the hydrolysate fractions and ascorbic acid (AA). All the values are mean ± SD. (a–g) Values with different letters indicate a significant difference at the same concentration for each group of samples (*p* > 0.05).

Free radical scavenging is a primary mechanism by which antioxidants inhibit oxidative processes. DPPH^•^ with a single electron exhibits modena and shows strong absorbance at 517 nm in ethanolic solution, and the absorbance reduces gradually when DPPH^•^ encounter a proton-donating substrate such as an antioxidant [21,34]. Figure 6A shows DPPH^•^ inhibition of the seven fractions at concentrations ranging from 0.2 to 5.0 mg/mL. All samples scavenged DPPH^•^ in a dose-dependent way at the tested concentrations. The DPPH^•^ scavenging ability of F7 was 65.72% ± 3.42% at a concentration of 5.0 mg/mL, and F1 showed the lowest DPPH^•^ scavenging activity (35.82% ± 1.65%) at this concentration. Samples with a lower AMW probably contain more peptides, which are electron donors that can react with free radicals to convert them to more stable products and terminate the radical chain reaction [21]. The EC_50_ of hydrolysate fractions F1 to F7 on DPPH^•^ scavenging activity were 8.95, 8.44, 7.62, 7.08, 4.51, 2.39 and 1.57 mg/mL, respectively. According to the literature [35,36,37,38], EC_50_ of F7 on DPPH^•^ scavenging ability was lower than those of protein hydrolysates from wheat germ (1.3 mg/mL), buckwheat (0.56–0.94 mg/mL), and rapeseed (0.45–0.6 mg/mL), but higher than those of protein hydrolysates from loach (2.64 mg/mL), hemp (2.3–6.2 mg/mL), abalone foot muscle (14.83 mg/mL), and scallop adductor muscle (10.27 ± 0.88 mg/mL), respectively. On the other hand, EC_50_ of F7 was significantly higher than that of the positive control of ascorbic acid (AA) (0.33 mg/mL).

As shown in Figure 6B, both samples and AA quenched HO^•^ in a concentration-dependent way in the tested concentration range. The HO^•^ scavenging ability of F7 was 76.99% ± 1.55% at concentration of 5.0 mg/mL, which was slightly lower than that of AA (81.64% ± 1.32%); by contrast, F1 showed the lowest HO^•^ scavenging ability (41.72% ± 2.87%). Just as the trend in DPPH^•^ scavenging activity, hydrolysate fractions with lower AMWs showed better scavenging abilities on HO^•^. The EC_50_ of hydrolysate fractions F1 to F7 were 6.59, 3.70, 3.24, 2.87, 2.61, 1.73 and 1.20 mg/mL, respectively. The HO^•^ scavenging abilities of the seven fractions were significantly higher than those of the protein hydrolysates from loach (17.0 mg/mL), abalone foot muscle (10.77 mg/mL), and scallop adductor muscle (13.76 mg/mL), respectively [39,40,41].

It is known that antioxidant activity is associated with the progress of reductones, which have been confirmed to be the terminators of chain reactions of free radical. Therefore, antioxidant activities of the seven ASC-SM hydrolysate fractions might be related to their reductive activities. Figure 6C indicates that the seven ASC-SM hydrolysate fractions own the capacity to reduce ferric ions to ferrous ions in a dose-dependent way. Among the seven hydrolysate fractions, lower AMWs samples revealed a stronger reducing power, but higher AMWs samples showed a weaker reducing power. The absorbance of F7 (5 mg/mL) at 700 nm was 2.87 ± 0.17, which was significantly higher than that of F1 (1.26 ± 0.65). The reducing power of the seven hydrolysate fractions was in the range of 1.26–2.87 at a concentration of 5 mg/mL, which was higher than those (0.08–1.24) of the protein hydrolysates from buckwheat, chickpea, and haemoglobin [36,40,41].

**Figure 7:**
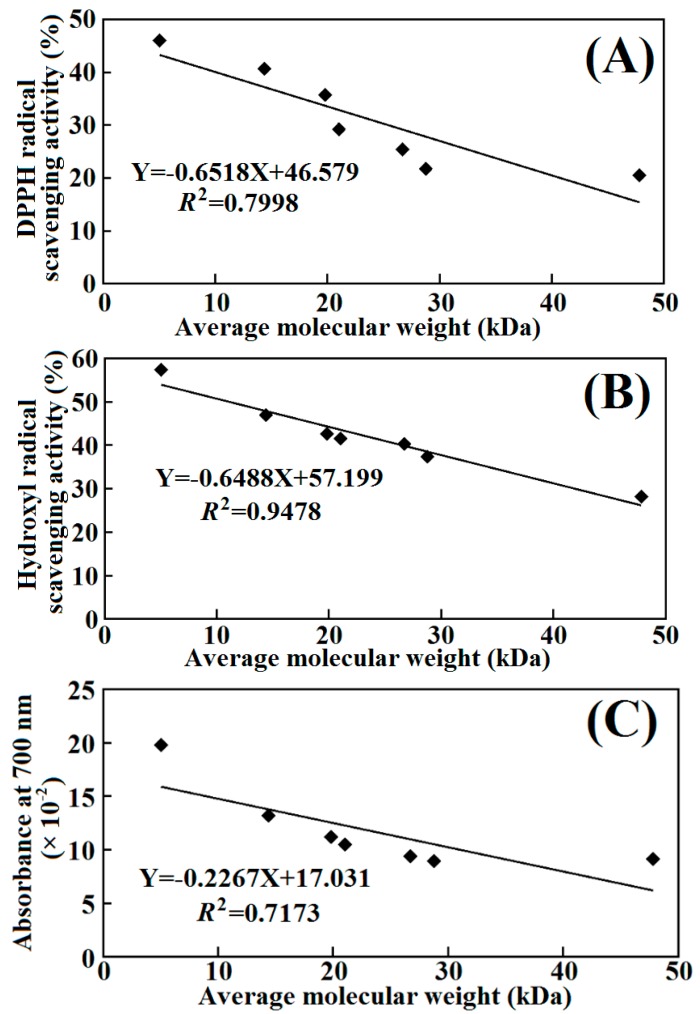
Correlations between antioxidant activities (DPPH^•^ scavenging activity (**A**), HO^•^ scavenging activity (**B**), and reducing power (**C**)) and AMWs of seven hydrolysate fractions. All the antioxidant activities are the mean values (*n* = 3).

As reported in the literature, the antioxidant capacities of the samples are closely associated with their abilities, such as deactiving reactive oxygen species (ROS), scavenging free radicals, chelating pro-oxidative transition metals, and donating electron/hydrogen [39]. The MW is believed to be one of the critical elements impacting on the antioxidant properties of protein hydrolysates [21,42,43]. In the present text, the correlation coefficients for antioxidant activities and AMWs of seven fractions were studied. As shown in Figure 7, the antioxidant activities of the seven fractions gradually reduced with the increase of their AMWs. The linear dependences between antioxidant activities and logarithms of AMWs were deduced, and showed noticeable negative correlations (from 0.7173 to 0.9478) between antioxidant activities and AMWs of hydrolysate fractions were found. The finding was in line with previous studies that report that high DH of hydrolysates was proven to be beneficial for their antioxidant activities [21,44,45]. Lower AMWs samples would be composed of shorter and more active peptides, which could serve as electron donors and react with free radicals to transform them into more stable substances and end the chain reactions. Moreover, samples with lower AMWs had a high probability to effectively traverse the intestinal barrier and fulfill their biological functions [18,22,46]. In the present research, the AMW was proven to be the key element that affected the antioxidant abilities of protein hydrolysates. 

### 2.7. Functional Properties

#### 2.7.1. Solubility

**Figure 8:**
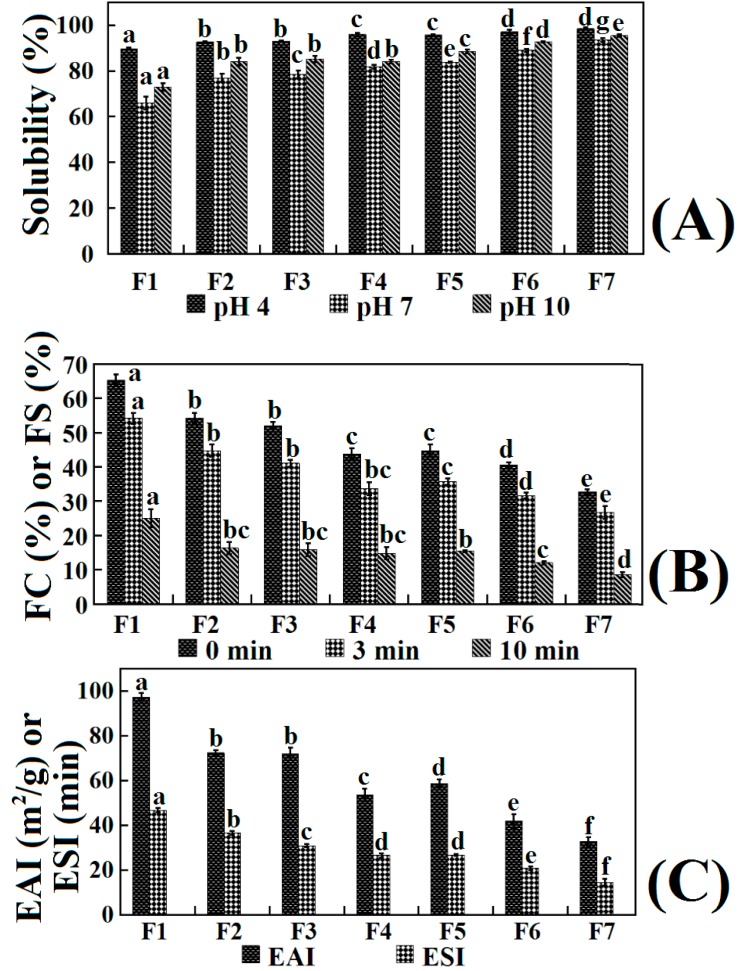
Solubility at different pH (**A**); foaming capacity (FC) or foam stability (FS) at different times (**B**); and emulsifying activity index (EAI) or the emulsion stability index (ESI) (**C**) of seven hydrolysate fractions. (a–g) Values with different letters indicate the significant difference for each group of samples under the same experimental conditions (*p* > 0.05).

The solubility profiles of the seven hydrolysate fractions (F1 to F7) are presented in Figure 8A. The result indicated that a decrease of AMWs could improve the solubility of samples at the tested pH values. Jridi *et al.* had reported that the increase in solubility of hydrolysates was due to the decrease of the MW and the release of smaller polypeptide fragments from the proteins [21]. The smaller peptides were expected to have proportionally more polar residues with the ability to form hydrogen bonds with water and increase solubility. However, the reduction in solubility of the larger AMW fractions could be explained by the fact that it was difficult for solvent molecules to surround the solute composed of larger molecules, which caused a decrease in number of hydrogen bonds, and further reduced the solubility of larger molecule substance.

On the other hand, pH had an obvious influence on the solubility of the ASC-SM hydrolysate fractions, especially on the lager AMW fractions. All of the seven fractions had a minimum and maximum solubility at pH 7 and pH 4, respectively. Taking F1 as an example, its solubility at pH 4, 7 and 10 was 89.71% ± 0.52%, 65.98% ± 2.76%, and 72.87% ± 1.65%, respectively. This finding was consistent with some research that had shown that collagen was easily dissolved in acidic water, but was hardly soluble in neutral solution [47]. It had been reported that the solubility of collagen could increase due to the repulsive force between chains when the net charge residues (negative or positive) increased if the pH was lower or higher than *pI*. The structure of fractions with higher AMW could be more similar to that of its parent collagen, which resulted in a more dramatic influence of different pH on their solubilities [24]. Hence, the desired collagen hydrolysates with excellent solubility could be prepared according to their AMWs.

#### 2.7.2. Foaming Properties

**Figure 9:**
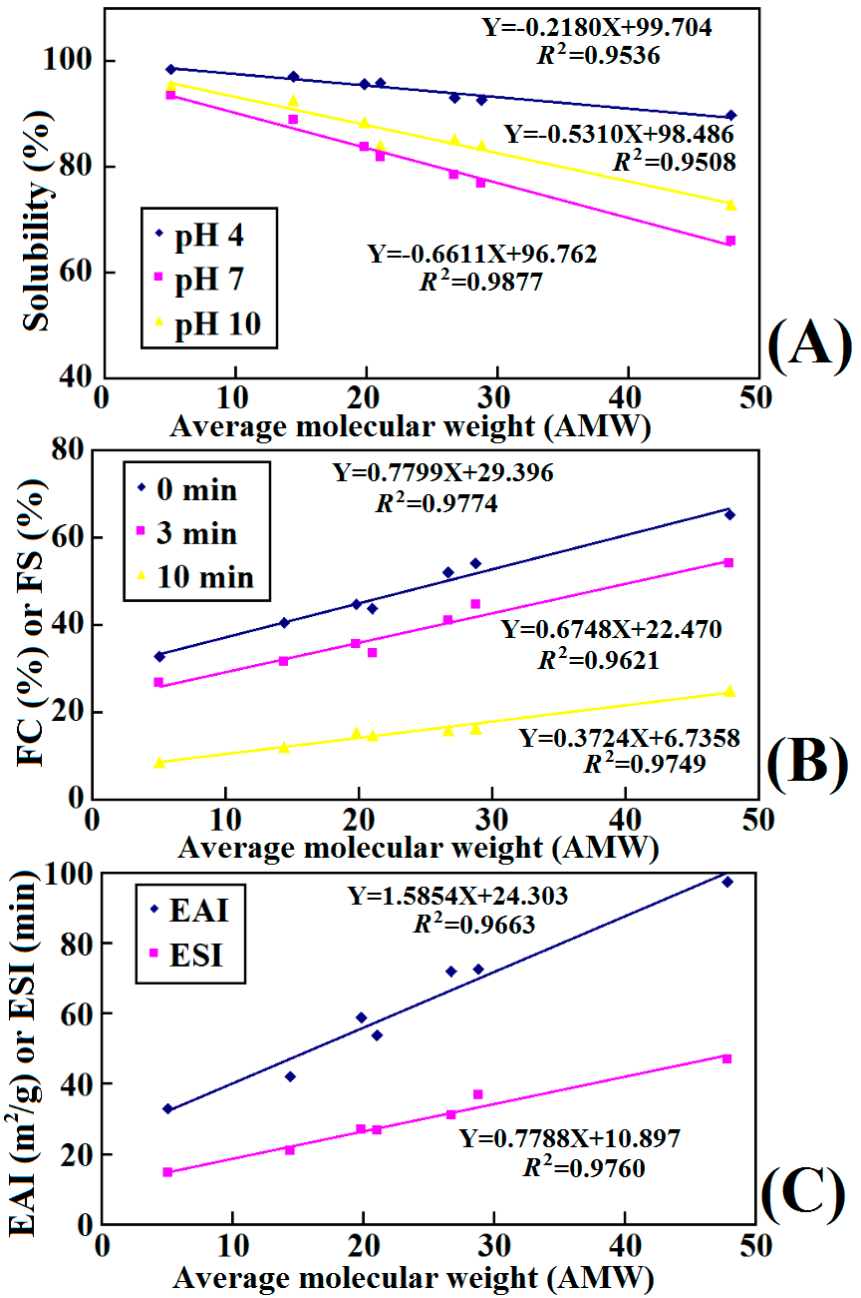
Correlations between functional properties (solubility (**A**), FC or FS (**B**), EAI or ESI (**C**)) and AMWs of seven hydrolysate fractions.

The foam capacity (FC) and foam stability (FS) of the seven hydrolysate fractions (F1 to F7) are shown in Figure 8B. The result indicated that the FC of F1 (65.27% ± 1.76%) was higher than those of the other six fractions (54.15%–32.76%) and protein hydrolysates from round scad (23.67%), sole (19%), and squid (18%) skins at the concentration of 0.5% [20,48]. In the same way, F1 showed higher FS (54.16 ± 1.65 and 25.04% ± 2.65% at time of 3 and 10 min) than those of the other six fractions (44.76–26.72 and 16.37%–8.55%, respectively). Compared with the SDS-PAGE pattern of lower AMW fractions (Figure 3), the hydrolysate fractions with higher AMW were composed of higher MW polypeptides, which could be beneficial to the formation of a stable film around the gas bubbles, which this might be the primary cause of their higher foaming properties. Using mathematical statistics, the linear relationship between AMWs and FC or FS was researched, and positive correlations (*R*^2^ ranging from 0.9621 to 0.9774) were observed (Figure 9B). The result suggested that collagen hydrolysates with high AMWs had better foaming properties than low AMW fractions. However, foaming abilities of collagen hydrolysates with high AMWs rapidly decreased after 10 min (Figure 8), and the duration time was shorter than that of common foaming agents. Therefore, more careful research is needed to study whether those collagen hydrolysates can be applied as foaming agents in food processing.

#### 2.7.3. Emulsifying Properties

Emulsifying activity index (EAI) and emulsion stability index (ESI) of the seven hydrolysate fractions (0.1%) are shown in Figure 8C. The result indicated that high AMWs fractions displayed higher EAI (97.44 ± 1.76 and 72.54 ± 1.05 for F1 and F2) than those (42.01 ± 2.98 and 32.87 ± 1.93 m^2^/g for F6 and F7) with low ones, and the ESI of the seven hydrolysate fractions showed a similar trend. Considering the seven fractions together, the dependency between AMW and EAI or ESI is shown in Figure 9C. The results indicated that there were positive correlations between AMWs and EAI or ESI. The finding was in line with the former report that larger polypeptides and collagens could be more effective to stabilize the protein film than small peptides in oil-in-water emulsion, and protein hydrolysates were surface-active materials and promoted an oil-in-water emulsion because of their hydrophilic and hydrophobic groups and their charges [21,36].

## 3. Experimental

### 3.1. Materials

The fresh Spanish mackerel (*S. niphonius*) was purchased from Nanzhen market in Zhoushan City, Zhejiang province of China, and authenticated by Prof. Sheng-long Zhao (Zhejiang Ocean University, Zhoushan, China).

### 3.2. Preparation of Acid Soluble Collagen (ASC) and Hydrolysates

ASC from Spanish mackerel skin was extracted by the previously described method and referred to as ASC-SM [26]. The ASC-SM solution (3.5 M) was prepared using acetic acid (0.5 M, pH 2.5). The mixture was incubated for 5 h at 37 °C after adding pepsin (2500–3500 U/mg protein, Sigma-Aldrich Trading Co., Ltd., Shanghai, China) with an enzyme/collagen ratio of 1:20 (w/w), heated to 90 °C and kept for 10 min in order to inactivate pepsin [49]. After that, the mixture was centrifuged at 5000 *×g* for 15 min at 4 °C, and the supernatant was freeze-dried and kept at −20 °C.

### 3.3. Fraction of ASC-SM Hydrolysate

Fifty milligrams of ASC-SM hydrolysate was dissolved in 5 mL of distilled water, loaded on Sephadex G-100 column (2.6 × 100 cm), and eluted using distilled water at a flow rate of 0.50 mL/min. Each eluted solution (5 mL) was collected and measured at 230 nm. The same fractions (F1 to F7) were pooled, concentrated, and lyophilized.

### 3.4. SDS-PAGE of ASC-SM and Its Hydrolysate Fractions

SDS-PAGE patterns were determined according to the previously described method [26], using 7.5% separating gel and 4% stacking gel for ASC-SM, and 12% separating gel and 5% stacking gel for its hydrolysate fractions. The sample was suspended in SDS solution (5% (w/v)) and incubated for 1 h at 85 °C. After that, the mixture was centrifuged at 5000 *×g* for 10 min to eliminate undissolved debris. The treated sample (20 μL) was mixed with the sample loading buffer at a ratio of 4:1 (v/v) in the presence of β-ME, added to sample wells and electrophoresed in an electrophoresis instrument of AE-6200 (Bio-sun Science and Technology Co., LTD., Shanghai, China). High and low MW protein markers (Shanghai Institute of Biochemistry, the Chinese Academy of Sciences, Shanghai, China) were applied to calculate the samples MW.

### 3.5. Proximate Analysis

Moisture, ash, fat, and protein contents of ASC-SM hydrolysate and its fractions were measured based on the methods of association of analytical communities (AOAC) (2003), and the phenol-sulfuric acid method was used to determine the polysaccharide contents of samples, using glucose as standard.

### 3.6. MW Distribution

MW distributions of ASC-SM hydrolysate fractions were determined by HPSEC on a TSK-G3000SW_XL_ column (Tosoh Co., Ltd., Shanghai, China) using a high-performance liquid chromatography (HPLC) system (Agilent 1260, Agilent Ltd., Santa Clara, CA, USA). The mobile phase was sodium phosphate buffer (0.1 M, pH 7.0). The sample (20 μL) was eluted at a flow rate of 0.5 mL/min and monitored at 230 nm. MW was measured using thyroglobulin (670 kDa), γ-globulin (150 kDa), ovalbumin (44 kDa), trypsin inhibitor (20.1 kDa), ribonuclease A (14.7 kDa), Pro-Tyr-Phe-Asn-Lys (667 Da) and Trp-Asp-Arg (475 Da) as references.

The linear equation between logMW and retention time (R_t_, min), as LogMW = −0.2036R_t_ + 7.6164 (*R*^2^ = 0.9766), was based on the method of least squares. The hydrolysate MW was calculated by R_t_. The sample chromatogram showed a continuous curve because samples differing by a few units could not be effectively separated, thus the peak area between the chromatogram curve and the baseline was vertically divided into slices at equal R_t_ (time interval 0.02 min). The percentage of slice *i* in peak area was calculated using Agilent HPLC workstation and referred to as *Pi* (%). The standard AMW of slice *i* was precisely calculated on the equation of LogMW = −0.2036R_t_ + 7.6164 and referred to as *Si* (kDa). Therefore, sample AMW was calculated on the Equation (1):

AMW = ∑^n^_i = 1_*Pi* × *Si* (kDa) (1)

### 3.7. Fourier Transform Infrared Spectroscopy (FTIR)

FTIR spectra (450–4000 cm^−1^) of ASC-SM and its hydrolysate fractions were recorded in KBr disks with a FTIR spectrophotometer of Nicolet 6700 (Thermo Nicolet Corporation, Madison, WI, USA). Dry sample was mixed with dry KBr at a ratio of 1:100 and pressed into a disk for spectrum recording. 

### 3.8. Scanning Electron Microscope (SEM)

Representative samples (ASC-SM, F1, and F7) were dissolved in acetic acid (0.5 M, 5% (w/v)), dialyzed against distilled water, and freeze-dried. The resulting samples were sputter coated for 90 s with gold and observed using a Scanning Electron Microscope (Hitachi TM-1000, Hitachi High-Technologies Corporation, Tokyo, Japan).

### 3.9. Antioxidant Activity

DPPH^•^ and HO^•^ scavenging activities and ferric ion reducing property were measured based on the methods of Li, Wang, Zhang, Qu, Xu, & Li [50].

#### 3.9.1. DPPH^•^ Scavenging Activity

Two milliliters of distilled water containing different concentrations of samples were placed in a cuvette, 500 μL of ethanol solution of DPPH (0.02%) and 1.0 mL of ethanol were added. The solution was incubated in the dark for 1 h and determined at 517 nm. A control sample containing DPPH without sample was also prepared. In blank, DPPH solution was substituted with ethanol. The antioxidant activity of the sample was evaluated by the inhibition percentage of DPPH with the following Equation (2):

Scavenging activity % = (A0 + A' − A)/A0 × 100%
(2)

Where A was the absorbance of the test sample; A0 was the absorbance of the control group; and A' was the absorbance of the blank.

#### 3.9.2. HO^•^ Scavenging Activity

In this system, HO^•^ was generated by the Fenton reaction. HO^•^ could oxidize Fe^2+^ into Fe^3+^, and only Fe^2+^ could be combined with 1,10-phenanthroline to form a red compound (1,10-phenanthroline-Fe^2+^) with the maximum absorbance at 536 nm. The concentration of HO^•^ was reflected by the degree of decolorization of the reaction solution. Briefly, 1,10-phenanthroline solution (1.0 mL, 1.865 mmol·L^−1^) and samples (2.0 mL) were added into a screw-capped tube in order and mixed homogeneously. The FeSO_4_•7H_2_O solution (1.0 mL, 1.865 mmol L^−1^) was then pipetted to the mixture. The reaction was initiated by adding 1.0 mL H_2_O_2_ (0.03% v/v). After incubation at 37 °C for 60 min in a water bath, the absorbance of reaction mixture was measured at 536 nm against the reagent blank. The reaction mixture without any antioxidant was used as the negative control, and the sample without H_2_O_2_ was used as the blank. The HO^•^ scavenging activity (HRSA) was calculated using the following formula (3):

HRSA (%) = [(As−An)/(Ab−An)] × 100
(3)

where As, An, and Ab are the absorbance values determined at 536 nm of the sample, the negative control, and the blank after reaction, respectively.

#### 3.9.3. Determination of Reducing Power

Two milliliters of each sample dissolved in distilled water was mixed with 2.5 mL of aqueous potassium hexacyanoferrate solution (1%). After being incubated at 50 °C for 30 min, 1.5 mL of trichloroacetic acid (10%) was added. Finally, 2.0 mL of the upper layer was mixed with 2.0 mL of distilled water and 0.5 mL of aqueous FeCl_3_ (0.1%), and the absorbance was recorded at 700 nm. Increased absorbance of the reaction mixture indicated increased reducing power.

### 3.10. Functional Properties

#### 3.10.1. Solubility

Solubility of ASC-SM hydrolysate fractions was detected based on the previously described method with slight modifications [51]. The lyophilized sample (200 mg) was dissolved in 20 mL of deionized water and the pH was adjusted to 4 or 7 or 10 with HCl (1 M) and/or NaOH (1 M). After that, the mixture was stirred for 30 min at 25 ± 1 °C and centrifuged at 10,000 *×g* for 15 min. The protein contents of supernatants and the overall suspensions were determined based on Lowry’s method using bovine serum albumin (BSA) as the standard. Protein solubility was calculated as the percent distribution of protein in the supernatant over the total protein content in the solution.

#### 3.10.2. Foaming Properties

FC and FS were measured based on the method of Shahidi *et al.* [24]. Sample solution (20 mL, 0.5%) was mixed with a cylinder (50 mL) at a speed of 16,000 *×g* to incorporate air for 2 min at 25 ± 1 °C. The total volume was detected at 0, 3 and 10 min after whipping. FC was defined as foam expansion at 0 min, and FS was defined as foam expansion during 10 min. Foam expansion was calculated based on the following Equation (4):

Foam expansion (%) = [(A − B)/B] × 100%
(4)

where A is the volume after whipping (mL) at different times and B is the volume before whipping (mL).

#### 3.10.3. Emulsifying Properties

EAI and ESI were calculated based on the method of Pearce and Kinsella [52]. Soybean oil (10 mL) and sample solution (30 mL, 0.1%) were mixed at a speed of 20,000 *×g* for 1 min, and the emulsion (50 μL) was pipetted from the container bottom at 0 and 10 min after homogenization and diluted 100-fold using 0.1% SDS solution. The absorbance of the diluted solution at 500 nm was determined using a spectrophotometer of UV-1800. The absorbances, determined immediately (*A_0_*) and 10 min (*A_10_*) after emulsion formation, were applied to calculate the EAI and the ESI as followed Equations (5) and (6):

EAI (m^2^/g) = (2 × 2.303 × *A*_500_)/(0.25 × protein concentration) (5)

ESI (min) = *A_0_* × △*t*/△*A* (6)

where *△*A = *A_0_* − *A_10_*, *△*t = 10 min.

### 3.11. Statistical Analyses

All statistical analyses were performed using SPSS 19.0 for windows (SPSS Inc., Chicago, IL, USA), and the value of *p* < 0.05 was used to indicate significant deviation detected by Duncan’s multiple range Test.

## 4. Conclusions

In the text, the relationships between the AMWs of ASC-SM hydrolysate fractions and their functional properties were researched. The increase of AMW considerably decreased the antioxidant activities and the solubility of hydrolysate fractions in the AMW range of 5.04–47.82 kDa, but an increase of AMW had positive influences mainly on the foaming and emulsifying capacities of the hydrolysate fractions. Based on the present results, the desired hydrolysates could be prepared using a controllable protease digestion process of the AMW.
